# Treatment Interval between Neoadjuvant Chemoradiotherapy and Surgery in Rectal Cancer Patients: A Population-Based Study

**DOI:** 10.1245/s10434-016-5294-0

**Published:** 2016-06-01

**Authors:** A. J. M. Rombouts, N. Hugen, M. A. G. Elferink, I. D. Nagtegaal, J. H. W. de Wilt

**Affiliations:** 1 Department of Surgery, Radboud University Medical Center, Nijmegen, The Netherlands; 2 Netherlands Comprehensive Cancer Organisation, Enschede, The Netherlands; 3 Department of Pathology, Radboud University Medical Center, Nijmegen, The Netherlands

## Abstract

**Background:**

Neoadjuvant chemoradiation therapy (CRT) has been widely implemented in the treatment of rectal cancer patients, but optimal timing of surgery after neoadjuvant therapy is unclear. The purpose of this study was to evaluate the effects of prolonged intervals between long-course CRT and surgery in rectal cancer patients.

**Methods:**

Data on all rectal cancer patients diagnosed between 2006 and 2011 were retrieved from the population-based Netherlands Cancer Registry; the main outcome parameters were pathologic complete response (pCR) and overall survival (OS). Outcomes were reported separately for patients with early tumors (ETs; *N* = 217) and locally advanced rectal cancer (LARC; *N* = 1073). Patients were divided into 2-week interval groups according to treatment interval, ranging from 5–6 to 13–14 weeks. Kaplan–Meier curves, and logistic regression and Cox regression models were used for data analysis.

**Results:**

No significant difference in pCR rate was observed for ET patients according to treatment interval. Compared with a treatment interval of 7–8 weeks, pCR rates in LARC patients were higher after 9–10 weeks (18.4 %; odds ratio [OR] 1.56, 95 % CI 1.03–2.37) and 11–12 weeks of treatment interval (20.8 %; OR 1.94, 95 % CI 1.15–3.26). Treatment interval did not influence OS in ET or LARC patients.

**Conclusions:**

Treatment intervals of 9–12 weeks between surgery and CRT seem to improve the chances of pCR in LARC patients, without an effect on OS. The length of treatment interval did not affect outcomes in patients with ET. The ongoing search for minimally invasive surgery drives the need for exploration of factors that improve pathologic response.

**Electronic supplementary material:**

The online version of this article (doi:10.1245/s10434-016-5294-0) contains supplementary material, which is available to authorized users.

In The Netherlands, rectal cancer is diagnosed in approximately 4500 patients annually.^1^ Total mesorectal excision (TME) is the gold standard in rectal cancer surgery and comprises resection of the rectal tumor together with the fatty tissue surrounding the rectum.^2^ Neoadjuvant treatment is used to improve outcome in case lymph node involvement is suspected or when the tumor extends to the mesorectal fascia on magnetic resonance imaging (MRI).^3^

For locally advanced rectal cancer (LARC), therapy consists of chemoradiation therapy (CRT) followed by TME or even beyond TME surgery.^4^ CRT consists of long-course radiotherapy of 45–50 Gy (in fractions of 1.8–2 Gy), with concurrent 5-fluorouracil. Treatment for early tumors (ETs) without suspicion of lymph node involvement (cT1–3N0) consists of TME surgery only; however, in a search for organ-sparing treatment modalities, CRT has also recently been applied to ET patients followed by local transanal excision.^5^–^11^ The wait-and-see approach has recently been introduced for both LARC and ET patients.^12^–^14^

Although neoadjuvant CRT has been widely implemented in the treatment of rectal cancer patients, the optimal timing of surgery after neoadjuvant therapy is as yet unclear.^15^ Surgery was previously performed 6–8 weeks after completion of CRT;^16^,^17^ however, recent studies suggest a time-related response of the tumor to CRT. There is growing evidence that extending the treatment interval might increase the proportion of patients with a complete pathologic response (pCR).^18^ It has also been suggested that a prolongation of the treatment interval might improve overall survival (OS).^18^,^19^ A meta-analysis on this topic did not show better OS in patients with longer treatment intervals, but the amount of evidence was scarce and the authors suggested further validation of the results.^18^

The currently available literature concerning the treatment interval between CRT and surgery does not differentiate between ET and LARC patients, despite the fact that there are substantial differences in oncological outcomes. This nationwide study aimed to evaluate the effects of prolonged treatment intervals on pCR and OS in ET and LARC patients separately.

## Methods

Data on all rectal cancer patients diagnosed between 2006 and 2011 in The Netherlands were retrieved from the population-based Netherlands Cancer Registry (NCR). The main source of notification of the NCR is the automated pathological archive (PALGA). Data were extracted from the medical records by trained registrars. Tumors are staged according to the TNM classification (5th edition) and classified according to the International Classification of Diseases for Oncology (ICD-O).

According to Dutch guidelines, all patients are staged using MRI.^17^ CRT is administered in patients with cT4 or cT3 and distance to the mesorectal fascia ≤1 mm, or cN2 and/or positive extramesorectal lymph nodes. All patients who were treated with neoadjuvant CRT followed by any kind of surgery were selected (*n* = 3042). Patients who were diagnosed with a tumor other than adenocarcinoma not otherwise specified (AC), mucinous adenocarcinoma, and signet ring cell adenocarcinoma were excluded (*n* = 30). Patients with metastatic disease (*n* = 249) or a treatment interval between CRT and surgery of <5 weeks or >15 weeks (*n* = 101) were also excluded since these were considered outliers (electronic supplementary Fig. 1). Missing data could not be retrieved retrospectively and patients for whom data concerning the time interval between CRT and surgery were unavailable were also excluded from the analysis (*n* = 1186). ETs were defined as a stage cT1-3N0 tumor (*n* = 217), and LARC was defined as a stage cT3Nx, cT3N1, cT4 and/or cN2 tumor (*n* = 1073). Patients with a cTx, cT1N1, or cT2N1 tumor (*n* = 186) did not meet the criteria of either of the groups and were not further analyzed.

pCR was defined as ypT0N0, and good pathologic response was defined as ypT0–T1N0. Registration of the circumferential resection margin (CRM) of the variables, and distance to anus in the cancer registry, was available from 2008. CRM was considered positive in case of a tumor-free resection margin ≤1 mm. The treatment interval was calculated from the end of CRT until the date of surgery, and follow-up data on vital status were retrieved by linkage to the nationwide municipal population registries network. Information concerning the cause of death was not available. Follow-up was calculated from the date of diagnosis to the date of death from any cause, or until 31 December 2011.

### Statistical Analysis

Statistical analyses were performed using SPSS software version 20.0 (IBM Corporation, Armonk, NY, USA). Baseline characteristics were analyzed using the *χ*^2^ test, or, in case of an expected cell count <5, the Fishers exact test or Monte Carlo simulation (number of samples 10,000) was used. OS was analyzed using Kaplan–Meier curves, and the log-rank test was used to compare survival curves. Patients who were alive at the end of follow-up were censored. Independent predictor variables for survival were estimated using Cox regression, and binary logistic regression was used to analyze predictors for pathologic response. Hazard ratios (HRs) resulting from Cox regression and odds ratios (ORs) resulting from logistic regression were reported alongside a 95 % confidence interval (CI). Variables to be taken into account in multivariable analyses were selected by significance during univariable analysis and clinical relevance; however, the variables of CRM and distance to anus were not included in the multivariable models because data were missing for patients before 2008. Reference groups for multivariable analyses were chosen based on the largest group size and results are only given in case of a *p* value of <0.1. Statistical significance was defined as *p* < 0.05.

## Results

The median interval between CRT and surgery was 8 weeks for ET and LARC patients. Baseline characteristics of patients included in the cohort were comparable with those of patients who were excluded because of unknown treatment interval and intermediate tumor stage (data not shown). Median follow-up was 50 months (range 5–106) in ET patients and 50 months (range 5–109) in LARC patients.

In ET patients, baseline characteristics were similar for groups divided according to treatment interval (Table 1), while in LARC patients, treatment intervals were longer for cT4 tumors (*p* = 0.007) and clinical node-positive tumors (*p* = 0.019; Table 2). The type of performed surgical procedure also differed between groups (*p* = 0.032); patients in the longer treatment interval groups more often underwent Hartmann procedures (Table 2).

**Table 1:** Characteristics for ET patients grouped according to interval between CRT and surgery

	5–6 weeks (*N* = 23)	7–8 weeks (*N* = 84)	9–10 weeks (*N* = 74)	11–12 weeks (*N* = 31)	13–14 weeks (*N* = 5)	*p* value
Age, years						0.639
Mean (range)	63 (45–79)	63 (34–85)	63 (36–84)	65 (39–84)	68 (62–76)	
<45	–	5 (6.0)	3 (4.1)	1 (3.2)	–	
45–59	8 (34.8)	19 (22.6)	24 (32.4)	5 (16.1)	–	
60–74	11 (47.8)	45 (53.6)	39 (52.7)	18 (58.1)	4 (80.0)	
>75	4 (17.4)	15 (17.9)	8 (10.8)	7 (22.6)	1 (20.0)	
Sex						0.549
Male	17 (73.9)	54 (64.3)	42 (56.8)	17 (54.8)	3 (60.0)	
Female	6 (26.1)	30 (35.7)	32 (43.2)	14 (45.2)	2 (40.0)	
Distance to anus, cm (*n* = 189)^a^						0.284
0–5	6 (42.9)	48 (64.0)	44 (67.6)	22 (73.3)	3 (60.0)	
6–10	7 (50.0)	20 (26.7)	10 (15.4)	5 (16.7)	2 (40.0)	
>10	1 (7.1)	5 (6.7)	5 (7.7)	1 (3.3)	–	
Unknown	–	2 (2.7)	6 (9.2)	2 (6.7)	–	
Histology						1.000
AC	21 (91.3)	77 (91.7)	68 (91.9)	29 (93.5)	5 (100.0)	
MC	2 (8.7)	6 (7.1)	6 (8.1)	2 (6.5)	–	
SRCC	–	1 (1.2)	–	–	–	
cT stage						0.927
cT1	–	–	–	–	–	
cT2	3 (13.0)	13 (15.5)	13 (17.6)	6 (19.4)	–	
cT3	20 (87.0)	71 (84.5)	61 (82.4)	25 (80.6)	5 (100.0)	
Surgical procedure						0.393
LAR	6 (26.1)	30 (35.7)	26 (35.1)	10 (32.3)	3 (60.0)	
APR	16 (69.6)	45 (53.6)	40 (54.1)	14 (45.2)	1 (20.0)	
Hartmann	1 (4.3)	5 (6.0)	3 (4.1)	2 (6.5)	1 (20.0)	
Other	–	4 (4.8)	5 (6.8)	5 (16.1)	–	
ypT stage						0.342
ypT0	8 (34.8)	12 (14.3)	18 (24.3)	4 (12.9)	2 (40.0)	
ypT1	2 (8.7)	8 (9.5)	6 (8.1)	6 (19.4)	–	
ypT2	4 (17.4)	29 (34.5)	18 (24.3)	11 (35.5)	–	
ypT3	9 (39.1)	33 (39.3)	28 (37.8)	10 (32.3)	3 (60.0)	
ypT4	–	–	–	–	–	
ypTx	–	2 (2.4)	4 (5.4)	–	–	
ypN stage						0.171
ypN0	19 (82.6)	70 (83.3)	55 (74.3)	23 (74.2)	3 (60.0)	
ypN1	3 (13.0)	8 (9.5)	6 (8.1)	5 (16.1)	2 (40.0)	
ypN2	1 (4.3)	4 (4.8)	4 (5.4)	–	–	
ypNx	–	2 (2.4)	9 (12.2)	3 (9.7)	–	
Differentiation						0.203
Well	–	–	–	2 (6.5)	–	
Intermediate	3 (13.0)	22 (26.2)	18 (24.3)	6 (19.4)	3 (60.0)	
Poor	–	5 (6.0)	3 (4.1)	2 (6.5)	–	
Unknown	20 (87.0)	57 (67.9)	53 (71.6)	21 (67.7)	2 (40.0)	

Data are expressed as *n* (%) unless otherwise specified

*AC* adenocarcinoma not otherwise specified, *MC* mucinous adenocarcinoma, *SRCC* signet ring cell adenocarcinoma, *LAR* low anterior resection, *APR* abdominoperineal resection, *ET* early tumor, *CRT* chemoradiation therapy

^a^Data were only available from 2008 and beyond

**Table 2:** Characteristics for LARC patients grouped according to interval between CRT and surgery

	5–6 weeks (*N* = 101)	7–8 weeks (*N* = 369)	9–10 weeks (*N* = 380)	11–12 weeks (*N* = 154)	13–14 weeks (*N* = 69)	*p* value
Age, years						0.127
Mean (range)	64 (31–82)	63 (32–85)	64 (27–83)	64 (39–82)	65 (42–83)	
<45	4 (4.0)	24 (6.5)	21 (5.5)	3 (1.9)	1 (1.4)	
45–59	30 (29.7)	100 (27.1)	115 (30.3)	47 (30.5)	17 (24.6)	
60–74	56 (55.4)	203 (55.0)	206 (54.2)	87 (56.5)	41 (59.4)	
>75	11 (10.9)	42 (11.4)	38 (10.0)	17 (11.0)	10 (14.5)	
Sex						0.907
Male	63 (62.4)	224 (60.7)	240 (63.2)	97 (63.0)	40 (58.0)	
Female	38 (37.6)	145 (39.3)	140 (36.8)	57 (37.0)	29 (42.0)	
Distance to anus, cm (*n* = 926)^a^						0.052
0–5	24 (42.1)	145 (46.6)	173 (48.7)	76 (54.3)	32 (50.8)	
6–10	19 (33.3)	125 (40.2)	130 (36.6)	37 (26.4)	25 (39.7)	
>10	8 (14.0)	24 (7.7)	37 (10.4)	14 (10.0)	1 (1.6)	
Unknown	6 (10.5)	17 (5.5)	15 (4.2)	13 (9.3)	5 (7.9)	
Histology						0.448
AC	96 (95.0)	335 (90.8)	341 (89.7)	134 (87.0)	63 (91.3)	
MC	5 (5.0)	33 (8.9)	37 (9.7)	19 (12.3)	5 (7.2)	
SRCC	–	1 (0.3)	2 (0.5)	1 (0.6)	1 (1.4)	
cT-stage						0.007
cT3	78 (77.2)	292 (79.1)	285 (75.0)	113 (73.4)	40 (58.0)	
cT4	23 (22.8)	77 (20.9)	95 (25.0)	41 (26.6)	29 (42.0)	
cN-stage						0.019
cN0	4 (4.0)	18 (4.9)	24 (6.3)	5 (3.2)	7 (10.1)	
cN1	64 (63.4)	193 (52.3)	171 (45.0)	67 (43.5)	35 (50.7)	
cN2	22 (21.8)	111 (30.1)	145 (38.2)	61 (39.6)	18 (26.1)	
cNx	11 (10.9)	47 (12.7)	40 (10.5)	21 (13.6)	9 (13.0)	
Surgical procedure						0.032
LAR	42 (41.6)	176 (47.7)	170 (44.7)	55 (35.7)	27 (39.1)	
APR	49 (48.5)	150 (40.7)	150 (39.5)	61 (39.6)	29 (42.0)	
Hartmann	5 (5.0)	23 (6.2)	25 (6.6)	20 (13.0)	7 (10.1)	
Other	5 (5.0)	20 (5.4)	35 (9.2)	18 (11.7)	6 (8.7)	
ypT stage						0.003
ypT0	8 (7.9)	52 (14.1)	78 (20.5)	35 (22.7)	13 (18.8)	
ypT1	5 (5.0)	16 (4.3)	23 (6.1)	12 (7.8)	2 (2.9)	
ypT2	23 (22.8)	103 (27.9)	79 (20.8)	28 (18.2)	13 (18.8)	
ypT3	57 (56.4)	171 (46.3)	166 (43.7)	62 (40.3)	27 (39.1)	
ypT4	7 (6.9)	17 (4.6)	22 (5.8)	14 (9.1)	11 (15.9)	
ypTx	1 (1.0)	10 (2.7)	12 (3.2)	3 (1.9)	2 (4.3)	
ypN stage						0.565
ypN0	64 (63.4)	237 (64.2)	244 (64.2)	101 (65.6)	45 (65.2)	
ypN1	25 (24.8)	80 (21.7)	81 (21.3)	33 (21.4)	14 (20.3)	
ypN2	12 (11.9)	51 (13.8)	49 (12.9)	19 (12.3)	7 (10.1)	
ypNx	–	1 (0.3)	6 (1.6)	1 (0.6)	3 (4.3)	
Differentiation						0.221
Well	2 (2.0)	5 (1.4)	5 (1.3)	–	3 (4.3)	
Intermediate	27 (26.7)	83 (22.5)	79 (20.8)	38 (24.7)	20 (29.0)	
Poor	4 (4.0)	17 (4.6)	25 (6.6)	13 (8.4)	5 (7.2)	
Unknown	68 (67.3)	264 (71.5)	271 (71.3)	103 (66.9)	41 (59.4)	

Data are expressed as *n* (%) unless otherwise specified

*AC* adenocarcinoma not otherwise specified, *MC* mucinous adenocarcinoma, *SRCC* signet ring cell adenocarcinoma, *LAR* low anterior resection, *APR* abdominoperineal resection, *LARC* locally advanced rectal cancer, *CRT* chemoradiation therapy

^a^Data were only available from the year 2008 and beyond

### Early Tumors

**Fig. 1:**
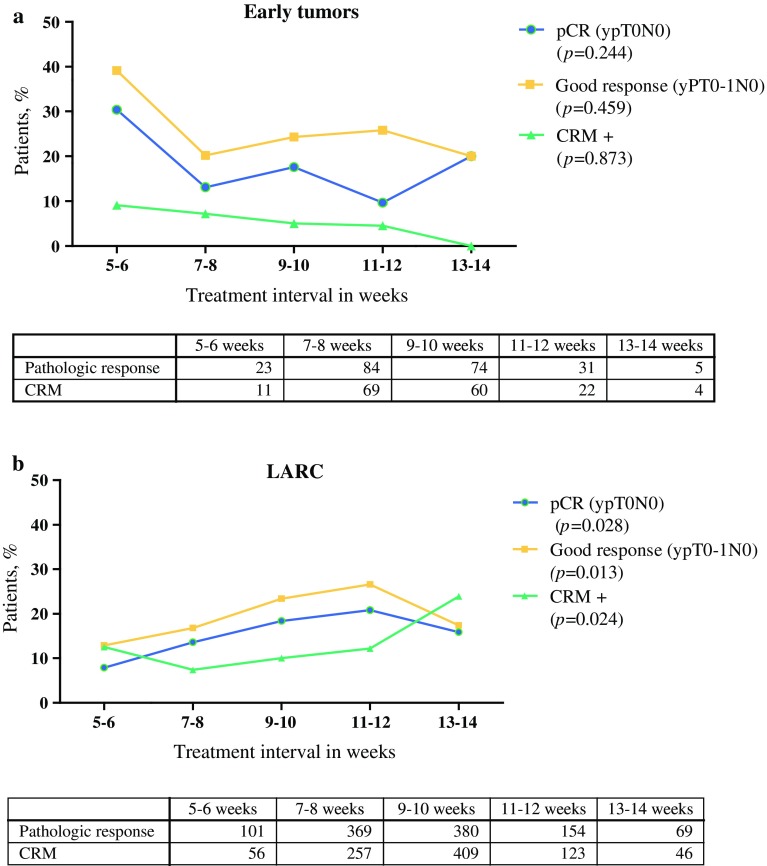
Percentage of patients with a pCR or good response, and percentage of patients with a positive CRM. **a** Patients with early tumors, **b** LARC patients. The tables show the number of patients in each group. For CRM, data were only available from 2008 and beyond. *CRM* circumferential resection margin, *pCR* pathologic complete response, *LARC* locally advanced rectal cancer

The overall pCR rate in ET patients was 16.1 %. The highest pCR rates and good response rates in ET patients were demonstrated after a treatment interval of 5–6 weeks (30.4 %, *n* = 7; and 39.1 %, *n* = 9, respectively; Fig. 1a), which was not significantly different in either univariable or multivariable logistic regression analysis (pCR, *p* = 0.148 [Electronic Supplementary Table S1]; good response, *p* = 0.541).

No relation was found between treatment interval and CRM involvement (Fig. 1a), ypT stage, or ypN stage (Table 1). Differences in treatment interval did not affect survival during either univariable or multivariable analysis (Electronic Supplementary Table S2). The relationship between pathologic response and OS was also analyzed, and patients with a complete or good pathologic response did not show an improved OS compared with other ET patients. In patients with a pCR, 5-year OS was 82.9 % (95 % CI 75.8–90.0) versus 75.6 % (95 % CI 71.6–79.6) in non-pCR patients (*p* = 0.332).

### Locally Advanced Rectal Cancer

The overall pCR rate in LARC patients was 15.9 %. pCR and good response rates were highest after an 11–12 week interval, with rates of 20.8 % (*p* = 0.028) and 26.6 % (*p* = 0.013), respectively (Fig. 1b). Outcomes of logistic regression are depicted in Table 3 and show that there was a higher odds of pCR in the 9- to 10-week and 11- to 12-week treatment interval groups than in the 7- to 8-week treatment interval group. This was also the case for good response rates (9–10 weeks: OR 1.67, 95 % CI 1.14–2.45, *p* = 0.008; and 11–12 weeks: OR 2.15, 95 % CI 1.34–3.48, *p* = 0.002). Other predictors for pCR in LARC patients were age, histology, and tumor differentiation (Table 3). Patients who were 45–59 years of age had lower pCR rates compared with patients who were aged between 60 and 74 years (OR 0.58, 95 % CI 0.38–0.88), and mucinous tumors were associated with lower pCR rates compared with patients with AC tumors (OR 0.19, 95 % CI 0.07–0.52; Table 3). pCR was more common in patients with an unknown tumor differentiation compared with patients with an intermediate tumor differentiation (OR 1.95, 95 % CI 1.25–3.05).

**Table 3:** Outcomes of multivariable logistic regression analysis of variables predicting pCR in LARC patients

	Odds ratio (95 % CI)	Adjusted *p* value
Treatment interval, weeks		0.022
5–6	0.57 (0.25–1.28)	0.172
7–8	1.00	–
9–10	1.56 (1.03–2.37)	0.037
11–12	1.94 (1.15–3.26)	0.013
13–14	1.44 (0.68–3.04)	0.346
Age, years		0.006
<45	1.78 (0.90–3.51)	0.099
45–59	0.58 (0.38–0.88)	0.011
60–74	1.00	–
>75	0.63 (0.35–1.15)	0.132
Histology		0.006
AC	1.00	–
MC	0.19 (0.07–0.52)	0.001
SRCC	1.19 (0.10–13.60)	0.887
Differentiation		0.014
Well	0.59 (0.07–4.76)	0.620
Intermediate	1.00	–
Poor	1.01 (0.36–2.80)	0.989
Unknown	1.95 (1.25–3.05)	0.003

Other variables entered into the model were sex, cT stage, cN stage, and year of surgery

*AC* adenocarcinoma not otherwise specified, *MC* mucinous adenocarcinoma, *SRCC* signet ring cell adenocarcinoma, *pCR* pathologic complete response, *LARC* locally advanced rectal cancer, *CI* confidence interval

The treatment interval in LARC patients was related to CRM involvement during univariable analysis (Fig. 1b), with the lowest rate of CRM-positive resections after a treatment interval of 7–8 weeks. Treatment interval did not affect ypN stage (*p* = 0.565). ypT0 was most prevalent in the 9- to 10-week (20.5 %) and 11- to 12-week (22.7 %) groups (*p* = 0.003). Longer treatment intervals did not affect survival (*p* = 0273; Table 4). Variables that did affect OS in LARC patients on multivariable analysis were pathologic response, age, sex and differentiation. LARC patients with a pCR had a better OS than patients who did not have a pCR: 88.9 % (95 % CI 86.3–91.5) of patients with pCR were alive after 5 years of follow-up compared with 71.0 % (95 % CI 69.3–72.7) of patients without pCR (HR 0.43, 95 % CI 0.27–0.68). Patients who were 75 years of age or older were associated with poorer survival compared with patients who were aged between 60 and 74 years (HR 2.0.1, 95 % CI 1.44–2.80). Patients who had a poorly differentiated tumor were associated with a poorer survival compared with patients whose tumor was intermediately differentiated (HR 2.43, 95 % CI 1.56–3.80). Female patients had better OS compared with male patients (HR 0.74, 95 % CI 0.57–0.96).

**Table 4:** Multivariable Cox regression analysis of variables predicting overall survival in LARC patients

	Hazard ratio (95 % CI)	Adjusted *p*-value
Treatment interval, weeks		0.273
5–6	1.39 (0.95–2.03)	0.095
7–8	1.00	–
9–10	0.99 (0.73–1.34)	0.935
11–12	1.13 (0.77–1.66)	0.543
13–14	1.44 (0.88–2.35)	0.144
pCR		<0.000
Yes	0.43 (0.27–0.68)	
No	1.00	
Sex		0.024
Male	1.00	
Female	0.74 (0.57–0.96)	
Age, years		<0.000
<45	0.67 (0.34–1.34)	0.259
45–59	0.91 (0.68–1.23)	0.548
60–74	1.00	–
>75	2.01 (1.44–2.80)	<0.000
Histology		0.093
AC	1.00	–
MC	1.28 (0.88–1.84)	0.197
SRCC	3.36 (0.96–11.79)	0.058
Differentiation		<0.000
Well	0.63 (0.20–2.03)	0.443
Intermediate	1.00	–
Poor	2.43 (1.56–3.80)	<0.000
Unknown	0.94 (0.70–1.26)	0.675

*pCR* pathologic complete response (ypT0N0), *AC* adenocarcinoma not otherwise specified, *MC* mucinous adenocarcinoma, *SRCC* signet ring cell adenocarcinoma, *LARC* locally advanced rectal cancer, *CI* confidence interval

## Discussion

Neoadjuvant CRT has been widely implemented in the treatment of rectal cancer to enable downsizing and downstaging and to improve outcome. In the present nationwide study, a treatment interval of 9–12 weeks led to the highest rates of pCR in LARC patients. This is a longer treatment interval than the usually reported interval in the literature, and half of the LARC patients in our cohort were operated within this 4-week interval. In ET patients, treatment interval did not seem to influence pCR rates. The length of treatment interval did not affect OS in either LARC or ET patients.

In addition to various important factors such as tumor stage, histology, and CRM,^20^ tumor response has been recognized as an important predictor of local recurrence and OS in rectal cancer surgery.^21^ It has repeatedly been suggested that the length of treatment interval between neoadjuvant therapy and surgery might influence pathologic response.^22^–^26^ To date, only two randomized controlled trials have been published comparing short and long treatment intervals between neoadjuvant therapy and surgery, and the outcomes are contradictory. The Lyon R90-01 trial (*N* = 201) in 1999 showed better pathologic tumor downstaging (T0–1) in a long interval group (6–8 weeks; 26 %) compared with a short interval group (2 weeks; 10.3 %; *p* = 0.005) between radiotherapy and surgery.^27^ In 2014, Saglam et al. compared outcomes for 4 or 8 weeks post-CRT (*N* = 153) and could not identify differences in complete response levels.^28^ A recent meta-analysis of all non-randomized trials on this topic (*N* = 3584) showed increased pCR rates after an interval of more than 6–8 weeks compared with pCR rates after a shorter treatment interval (from 13.7 to 19.5 %).^24^ This corroborates well with the findings in the current cohort of LARC patients.

Other factors that were identified as predictors of pathologic response in LARC patients were age, tumor differentiation, and mucinous histology. The latter is in accordance with a recent study from our group that reported on outcomes in LARC patients with mucinous AC and AC. This study demonstrated that pCR was not observed in any mucinous AC patient compared with over 16 % in AC patients, which did not affect survival.^29^ In the current cohort, pCR rates were again significantly lower in mucinous AC patients without an effect on OS. A recent study of the National Cancer Data Base (NCDB) (*N* = 23,747) identified a longer treatment interval, lower tumor grade, lower cT and cN stage, and higher radiation dose as predictors for pCR.^26^ Other studies did not identify any independent determinants in achieving pCR other than treatment interval.^22^,^23^

Outcomes for ET patients were analyzed separately in the current study and the treatment interval in these patients was not related to pathologic response. As far as we know, no other study to date has analyzed the effects of treatment interval in a select group of ET patients. The observed overall pCR rate in ET patients in the current study was 16.1 %, which is substantially lower than reported pCR rates in recent literature of 22–44 %.^6^,^8^,^9^,^11^,^30^ Several issues for this discrepancy can be noted. First, most of these studies with higher pCR rates are conducted prospectively and patient selection might explain the high response rates. Furthermore, in the present study, details on clinical staging could not be checked, and stages could have been higher. Also, the group of ET patients is probably biased since these patients received CRT, although this is not generally recommended according to national guidelines. Larger studies are needed to assess whether the duration of treatment interval influences pathologic response in ET patients and could reduce the need for surgery.

Treatment interval was not related to OS in either ET or LARC patients. Only a limited number of studies have demonstrated improved survival for LARC patients with a long treatment interval.^15^,^31^ To the best of our knowledge, none of the studies to date that have analyzed the relationship between a prolonged treatment interval and OS have specifically looked into outcomes of ET patients.^15^,^25^,^28^,^31^–^33^

The present study describes a population-based analysis of a large number of rectal cancer patients, however it has some limitations. The retrospective nature of this study poses particular caution because of heterogeneity in CRT regimens and other potential confounding variables. For example, reasons for either a short or longer treatment interval could not be retrieved. It is not inconceivable that patient or tumor characteristics have influenced the timing of surgery. In addition, 1186 patients had to be excluded from analysis because the length of their treatment interval was unknown. This could have introduced a selection bias, but comparison of this group with the included patients in the study did not show differences in characteristics. Randomized trials are necessary to determine the optimal treatment interval following CRT. Several trials are ongoing at the moment and are evaluating treatment intervals of 6–7 weeks versus 11–12 weeks following neoadjuvant CRT.^34^–^36^

Promising outcomes have been reported in studies analysing different treatment modalities, including a wait and see approach^12^,^14^ and local transanal excision.^5^–^11^ Oncological outcomes have been shown to be comparable with those of patients with a pCR after major surgery.^5^,^6^,^11^,^12^,^14^ Moreover, a reduction in the number of complications and permanent stomas might lead to a better functional outcome;^12^ however, these outcomes still have to be confirmed in randomized trials. Another drawback of the rectum-saving approach is that patients who need completion surgery will be overtreated with CRT, which has potential and serious side effects and long-term complications.^7^,^14^ It is therefore necessary that selection criteria of eligible patients be further explored prior to implementation.

## Conclusions

Treatment intervals of 9–12 weeks seem to improve the chances of pCR in LARC patients, without an effect on OS. The optimal treatment interval between surgery and CRT for ET patients could not be identified. The ongoing search for minimally invasive surgery drives the need for exploration of factors that improve pathologic response.

## Electronic supplementary material

Below is the link to the electronic supplementary material.

Supplementary material 1 (DOCX 75 kb)
