# Supplementary material for: Treatment Interval between Neoadjuvant Chemoradiotherapy and Surgery in Rectal Cancer Patients: A Population-Based Study
Source: Ann Surg Oncol. 2016 Jun 1;23(11):3593–601. doi: 10.1245/s10434-016-5294-0 (PMC5009153; doi:10.1245/s10434-016-5294-0)
Supplement: Supplementary file 1 — Supplementary material 1 (DOCX 75 kb) [file 10434_2016_5294_MOESM1_ESM.docx]

## Supplementary tables and figures

**Supplementary table S1** Multivariable logistic regression analysis of variables predicting pCR in patients with early tumors

|  | **Odds ratio**  **(95% CI)** | **Adjusted *p*-value** |
| --- | --- | --- |
| **Treatment interval**  5-6 wks  7-8 wks  9-10 wks  11-12 wks  13-14 wks | 4.26 (1.10-16.56)  1.00  1.25 (0.49-3.20)  0.42 (0.08-2.16)  2.21 (0.20-24.38) | 0.148  0.036  -  0.642  0.300  0.516 |

*Values in parentheses are 95% confidence intervals. Other variables entered into the model were: age, surgical procedure, histology, differentiation, clinical tumor stage and year of surgery.*

**Supplementary table S2** Multivariable Cox regression analysis of variables predicting OS in patients with early tumors

|  | **Hazard ratio**  **(95% CI)** | **Adjusted *p*-value** |
| --- | --- | --- |
| **Treatment interval**  5-6 wks  7-8 wks  9-10 wks  11-12 wks  13-14 wks | 0.57(0.20-1.59)  1.00  0.67 (0.31-1.47)  0.82 (0.31-2.17)  1.33 (0.15-12.21) | 0.760  0.281  -  0.322  0.683  0.800 |
| **pCR**  yes  no | 0.876(0.33-2.36)  1.00 | 0.793 |
| **Age**  <45  45-59  60-74  >75 | 1.50 (0.33-6.83)  1.13 (0.49-2.65)  1.00  4.02 (1.85-8.74) | 0.003  0.602  0.772  -  <0.000 |
| **Histology**  AC  MC  SRCC | -  2.34 (0.77-7.11)  15.54 (1.77-136.27) | 0.019  -  0.135  0.013 |

*Values in parentheses are 95% confidence intervals. pCR, complete pathologic response (ypT0N0); AC, adenocarcinoma not otherwise specified; MC, mucinous adenocarcinoma; SRCC, signet-ring cell adenocarcinoma. Other variables entered into the model were: sex, adjuvant chemotherapy, , differentiation, surgical procedure and year of surgery.*


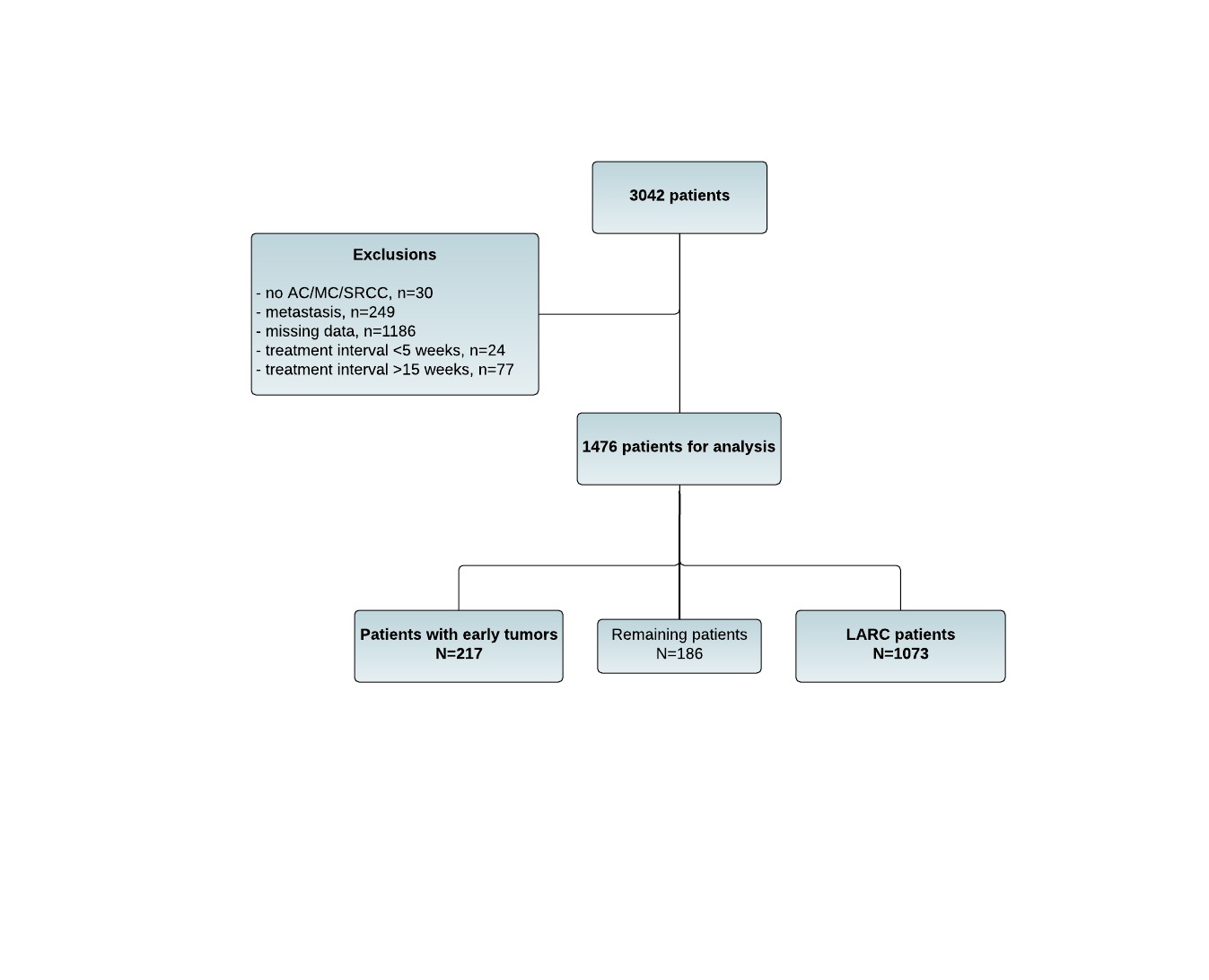


**Supplementary fig. S1** Study flow diagram. AC, adenocarcinoma not otherwise specified; MC, mucinous adenocarcinoma; SRCC, signet-ring cell adenocarcinoma; LARC, locally advanced rectal cancer. Remaining patients concern those with a with a cTx, cT1N1 or cT2N1 tumor and were not further analysed.
